# Biallelic *POC1A* variants cause syndromic severe insulin resistance with muscle cramps

**DOI:** 10.1530/EJE-21-0609

**Published:** 2022-03-01

**Authors:** Veronica Mericq, Isabel Huang-Doran, Dhekra Al-Naqeb, Javiera Basaure, Claudia Castiglioni, Christiaan de Bruin, Yvonne Hendriks, Enrico Bertini, Fowzan S Alkuraya, Monique Losekoot, Khalid Al-Rubeaan, Robert K Semple, Jan M Wit

**Affiliations:** 1 Institute of Maternal and Child Research, Faculty of Medicine, University of Chile, Santiago, Chile; 2 Department of Pediatrics, Clinica Las Condes, Santiago, Chile; 3 University of Cambridge Metabolic Research Laboratories and NIHR Cambridge Biomedical Research Centre, Wellcome Trust-MRC Institute of Metabolic Science, Addenbrooke’s Hospital, Cambridge, UK; 4 Department of Medicine, Medical Genetic Clinic, Sultan Bin Abdulaziz Humanitarian City, Riyadh, Saudi Arabia; 5 Complejo Asistencial Dr. Sotero del Rio, Santiago, Chile; 6 Department of Pediatric Neurology, Clinica Las Condes, Santiago, Chile; 7 Division of Paediatric Endocrinology, Department of Paediatrics, Willem-Alexander Children’s Hospital, Leiden University Medical Center, Leiden, Netherlands; 8 Department of Clinical Genetics, Leiden University Medical Centre, Leiden, Netherlands; 9 Unit of Neuromuscular and Neurodegenerative Disorders, Genetics and Rare Diseases Research Division, Bambino Gesù Children’s Hospital, IRCCS, Rome, Italy; 10 Department of Translational Genomics, Centre for Genomic Medicine, King Faisal Specialist Hospital and Research Centre, Riyadh, Saudi Arabia; 11 Research and Scientific Centre Director, Sultan Bin Abdulaziz Humanitarian City, Riyadh, Saudi Arabia; 12 Center for Cardiovascular Science, University of Edinburgh, Edinburgh, UK

## Abstract

**Objective:**

To describe clinical, laboratory, and genetic characteristics of three unrelated cases from Chile, Portugal, and Saudi Arabia with severe insulin resistance, SOFT syndrome, and biallelic pathogenic *POC1A* variants.

**Design:**

Observational study.

**Methods:**

Probands’ phenotypes, including short stature, dysmorphism, and insulin resistance, were compared with previous reports.

**Results:**

Cases 1 (female) and 3 (male) were homozygous for known pathogenic *POC1A* variants: c.649C>T, p.(Arg217Trp) and c.241C>T, p.(Arg81*), respectively. Case 2 (male) was compound heterozygous for p.(Arg217Trp) variant and the rare missense variant c.370G>A, p.(Asp124Asn). All three cases exhibited severe insulin resistance, acanthosis nigricans, elevated serum triglycerides and decreased HDL, and fatty liver, resembling three previously reported cases. All three also reported severe muscle cramps. Aggregate analysis of the six known cases with biallelic *POC1A* variants and insulin resistance showed decreased birth weight and length mean (s.d.): −2.8 (0.9) and −3.7 (0.9) SDS, respectively), severe short stature mean (s.d.) height: −4.9 (1.7) SDS) and moderate microcephaly (mean occipitofrontal circumference −3.0 (range: −4.7 to −1.2)). These findings were similar to those reported for patients with SOFT syndrome without insulin resistance. Muscle biopsy in Case 3 showed features of muscle involvement secondary to a neuropathic process.

**Conclusions:**

Patients with SOFT syndrome can develop severe dyslipidaemic insulin resistance, independent of the exonic position of the *POC1A* variant. They also can develop severe muscle cramps. After diagnosis, patients should be regularly screened for insulin resistance and muscle complaints.

## Introduction

SOFT syndrome (MIM # 614813), denoting short stature, onychodysplasia, facial dysmorphism, and hypotrichosis, is the name coined for a rare primordial dwarfism syndrome encompassing severe growth failure of prenatal onset, craniofacial dysmorphism, sparse hair, and digital abnormalities (1). In 2012, two groups reported that the syndrome was caused by biallelic variants in *POC1A*, encoding the proteome of centrioles 1A (POC1A) protein (1, 2, 3). POC1A is an important luminal component of centrioles, playing roles in the function of centrosomes, spindle poles, and ciliary basal bodies (4, 5, 6).

Since these initial reports (1, 2, 3), 12 additional affected kindreds have been described (7, 8, 9, 10, 11, 12, 13, 14, 15, 16). In addition to the cardinal syndromic features, three of 31 patients reported to date also manifested severe dyslipidaemic insulin resistance (IR) (7, 11, 16). All 3 harboured pathogenic variants in exon 10, raising the possibility of a distinct, exon-specific ‘variant POC1A-related’ (vPOC1A) subsyndrome (11). However, an exon 9 variant in the most recently reported patient with IR (16), and variants outside exon 10 in two further individuals with early-onset type 2 diabetes (DM2) (2), suggest that IR may be part of the wider SOFT syndrome phenotype, and not uniquely associated with exon 10 variants.

We now present clinical, biochemical, and genetic characteristics of three unrelated patients carrying biallelic pathogenic *POC1A* variants outside exon 10 who show clinical features of SOFT syndrome plus severe dyslipidaemic IR, providing further evidence that severe IR with or without DM2 is a frequent component of SOFT syndrome. All three also suffer from severe muscle spasms and cramps, reported only in one patient to date (2).

## Subjects and methods

### Study approval

**Figure 1:**
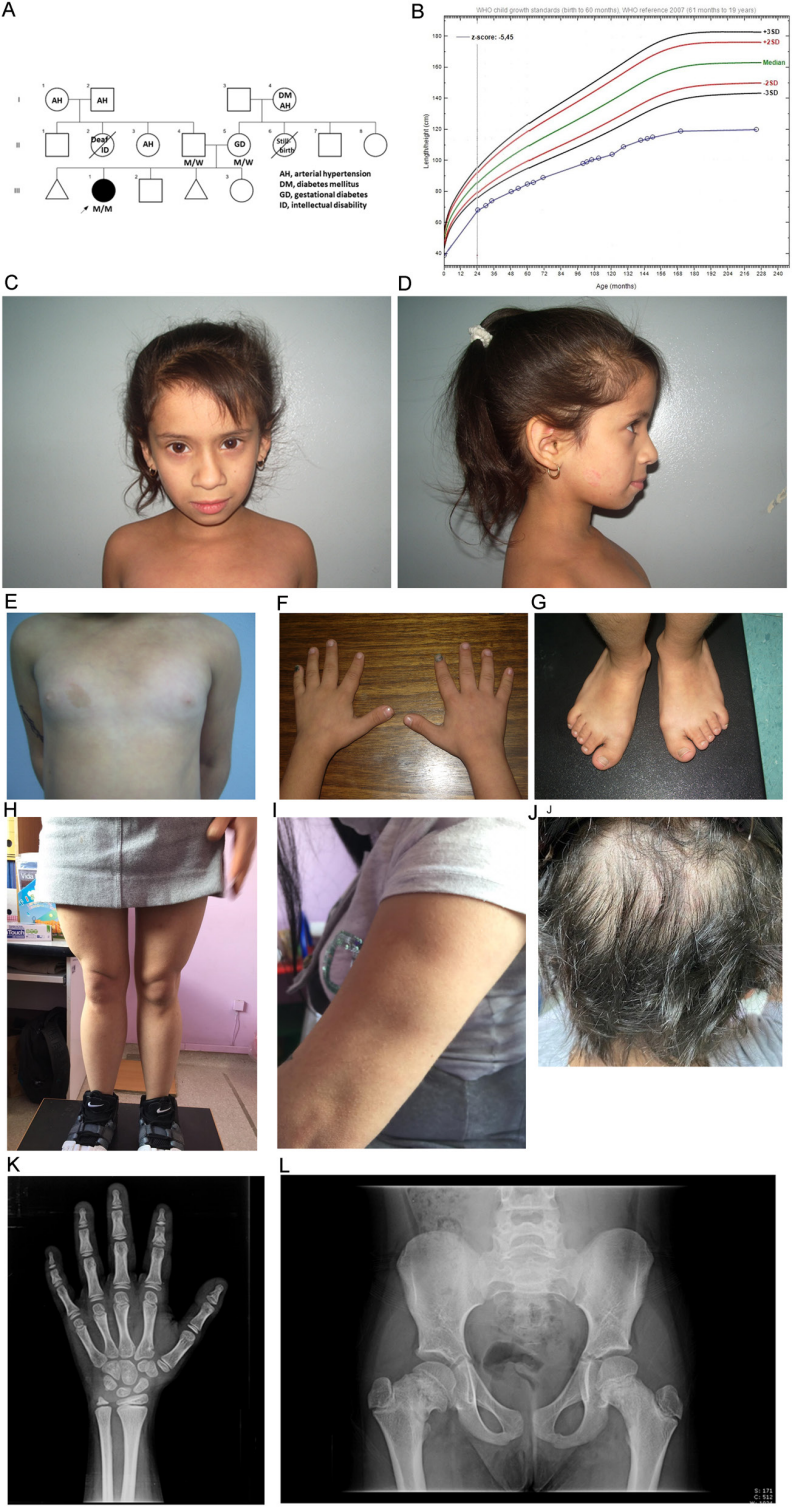
Case 1. (A) Pedigree (using INVITAE Family pedigree tool). M/M indicates a biallelic *POC1A* variant, M/W a heterozygous carrier. (B) Growth curve (height for age) against CDC chart. (C and D) Frontal and lateral photographs aged 8.8 years. (E) Chest at 8.8 years showing the café au lait spot. (F) Hands show brachydactyly and mild fifth finger clinodactyly and broad thumbs. The nails were broad and short. (G) Feet show broad big toes. (H) Broad upper legs. (I) Muscle cramps aged 21.5 years. (J) Scalp aged 21.5 years. (K) The hand X-ray aged 8.7 years shows short phalanges, cone epiphyses of the distal phalanges, pseudo-epiphysis in the middle phalanx of the index, clinodactyly of the little finger, and a slight delay in bone maturation. (L) The pelvic X-ray aged 8.7 years shows asymmetric involvement of the femoral necks with abnormal remodelling, shortening, and deformity.

**Figure 2:**
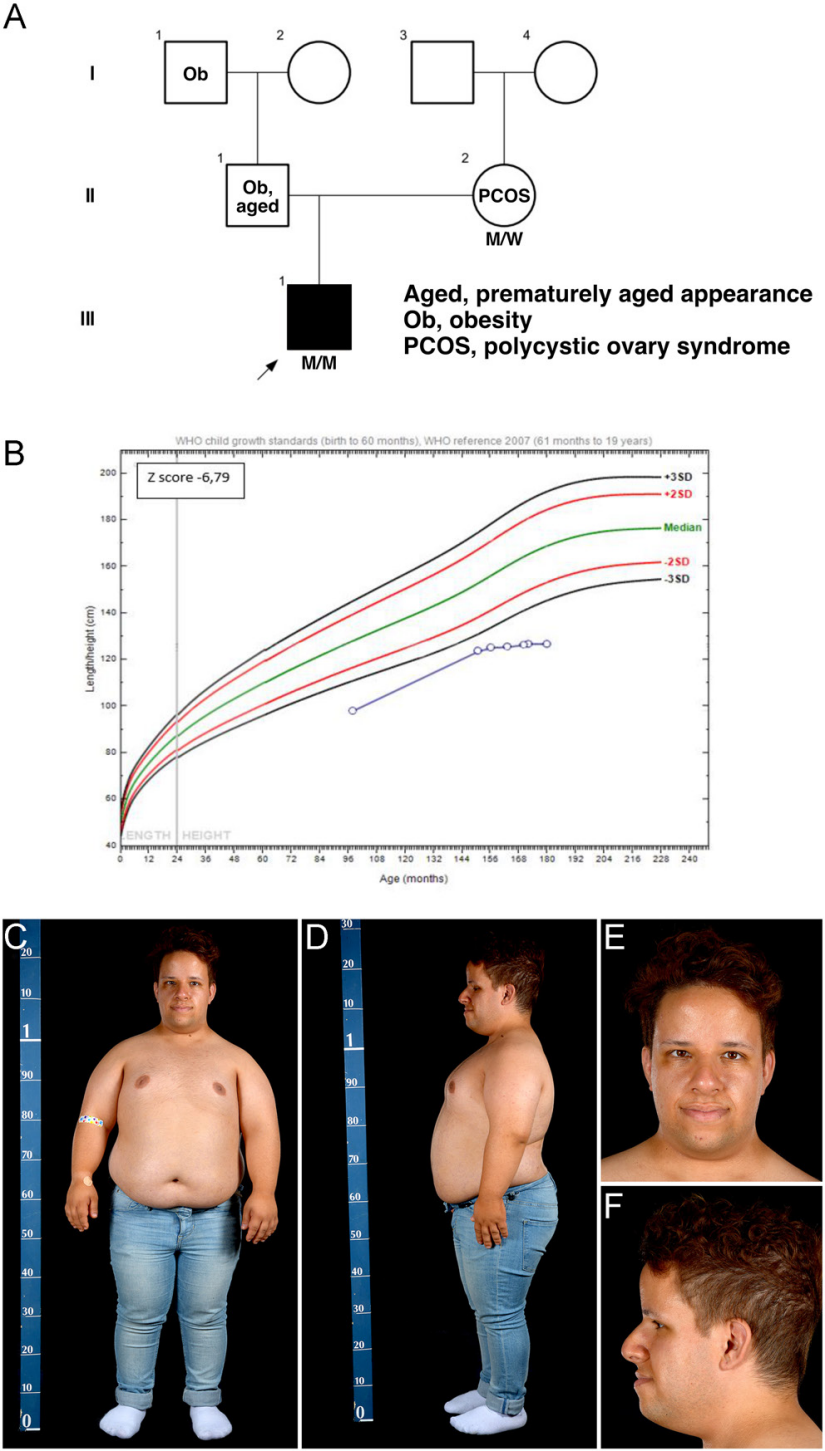
Case 2. (A) Pedigree (using INVITAE Family pedigree tool). M/M indicates a biallelic *POC1A* variant, M/W a heterozygous carrier. (B) Height plotted against CDC charts. (C, D, E, and F) Frontal and lateral photographs aged 22.3 years.

**Figure 3:**
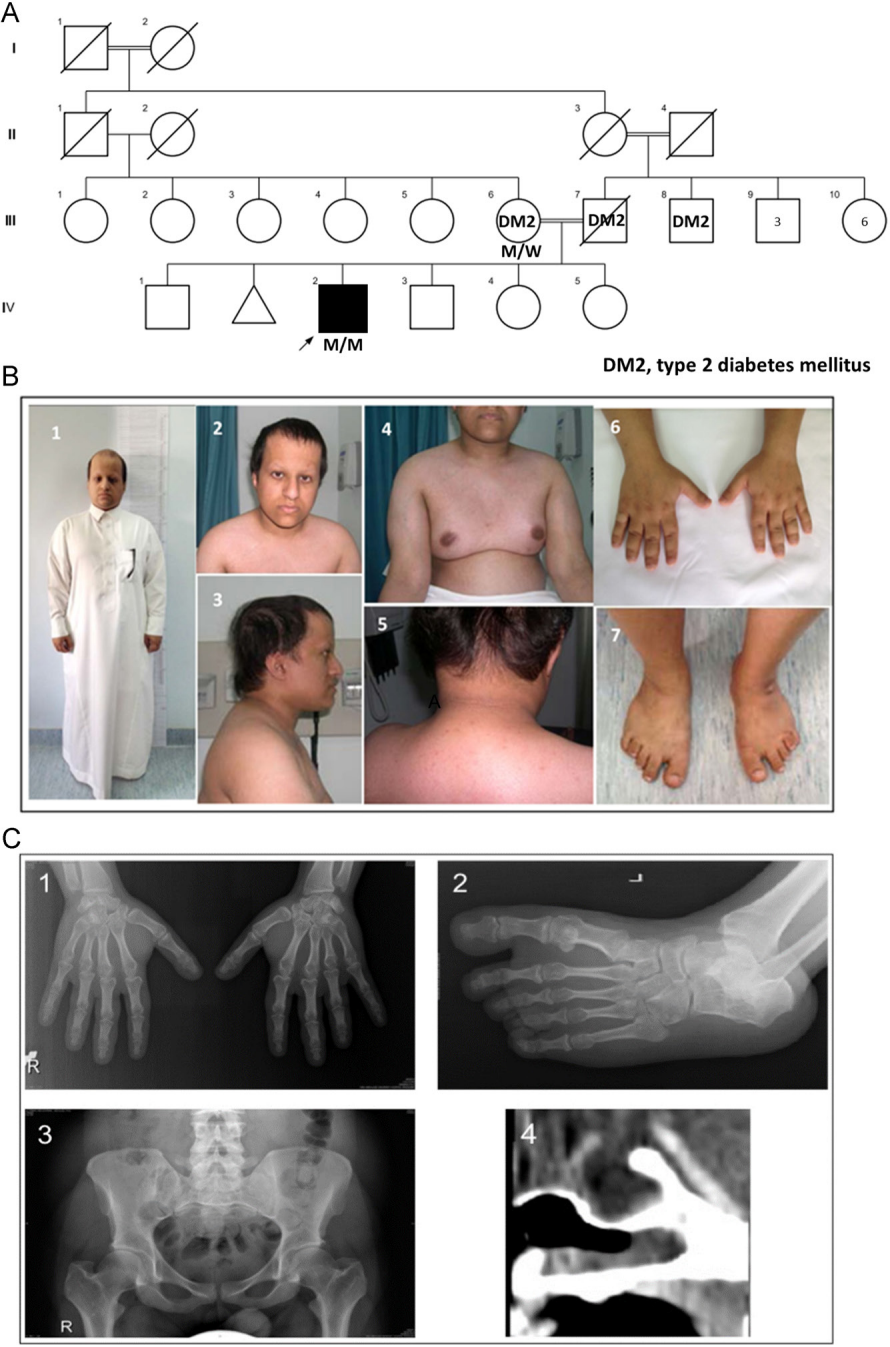
Case 3. (A) Family pedigree (using INVITAE Family pedigree tool). M/M indicates a biallelic POC1A variant, M/W a heterozygous carrier. (B) Clinical features demonstrating the abnormal findings: (i) short stature; (ii) high forehead and frontal bossing; (iii) posterior low set ear; (iv) gynaecomastia; (v) Acanthosis nigricans; (vi) hypoplastic distal phalanges and nails; (vii) wide space between big and second toes. (C) Radiological abnormalities: (i) short third metacarpal; (ii) metatarsal bone; (iii) short femoral neck; (iv) empty sella turcia.

Patients were enrolled in genetic research projects or were referred for diagnostic genetic testing. All investigations were conducted according to the Declaration of Helsinki principles. Clinical data and images were collected with signed informed consent from participants/families. Permission was obtained to publish images in Figs 1, 2, 3, and Supplementary Fig. 1 (see section on supplementary materials given at the end of this article).

### Case reports

Detailed clinical information on the three cases is presented in the supplementary information on clinical presentations and their developmental history, clinical history, and physical examination findings are summarised in Table 1.

**Table 1:** Developmental history, clinical history, and physical examination findings in the three cases.

Features	Case 1	Case 2	Case 3
Development			
Gender	Female	Male	Male
Current age	21 years	25 years	32 years
Parents	Reportedly unrelated	Not related	First cousin consanguineous
Birth weight	1520 g (−4.4 SDS)	2450 g (−3.2 SDS)	1800 g (−2.8 SDS)
Birth length	39 cm (−5.5 SDS)	NR	45 cm (−3.0 SDS)
Birth OFC	31 cm (−2.4 SDS)	NR	33 cm (−1.2 SDS)
Psychomotor development	Normal	Normal	Delayed
Linear growth	Severe growth failure. Adult height 120 cm (−6.6 SDS)	Severe growth failure. Adult height 127 cm (−7.2 SDS)	Severe growth failure. Adult height 138 cm (−5.8 SDS)
Clinical observations			
Insulin resistance	Insulin resistance which progressed to type 2 diabetes	Insulin resistance with reactive hypoglycaemia	Insulin resistance which progressed to type 2 diabetes
Hypertension	Present, treated	NR	Absent
Hyperlipidaemia	Diagnosed at 11 years	Diagnosed at 22 years	Diagnosed at 22 years
Ophthalmological assessment	Astigmatism	NR	Mild non-proliferative diabetic retinopathy
Pubertal development	Tanner B2 at 9.8 years, menarche at 15.3 years	Tanner G2 at 11 years; G3 (testes 8 mL) at 13.5 years	Absent (G1 at 21 years), gynaecomastia
Muscle cramps	Onset aged 2 years	Onset aged 13 years	Onset aged 22 years
Alopecia	Present	Present	Present
Centripetal obesity	Absent (waist circumference 72 cm)	Present	Present
Acanthosis Nigricans	Present from 10.1 years	Present from 13.5 years	Present from 21 years
Hypotonia	NR	NR	Present
High pitched voice	Present	Present	Absent
Adult gonadal status	Partial ovarian failure	NR	Borderline low plasma testosterone
Laboratory			
Insulin	Increased	Increased	Increased
Creatine Kinase	Increased	Increased	Increased
Additional findings			
Empty sella turcica	NR	NR	Present
Diffuse fatty liver	Present	Present	Present
Kidney anatomy	Normal kidney ultrasonography	NR	Left ectopic kidney
Electromyography	Reduced recruitment of MUAPs firing at increased frequency with increased amplitude, polyphasic potentials. Spontaneous fasciculations.	NR	Rare fibrillations and positive sharp waves. Normal MUAPs, morphology and recruitments. Muscular cramps induced by leg exercise accompanied by fasciculation
Colonoscopy	NR	NR	Transverse colon polyp, no dysplasia or malignancy

MUAPs, motor unit action potentials; NR, not reported; OFC, occipitofrontal circumference; SDS, standard deviation score.

#### Case 1

*Case 1* is a 21.5-year-old Chilean woman born to healthy parents of normal height (Fig. 1A) with an extremely low birth size and poor postnatal growth (Fig. 1B and Table 1). Further clinical features include microcephaly, bilateral hip pain, prominent forehead, deep-set eyes, hypoplastic nostrils, smooth philtrum, thin upper lip, light skin, café au lait macules, joint hyperlaxity, broad hands and feet with broad thumbs/big toes, and broad upper legs (Fig. 1C, D, E, F, G, and H). Radiographs showed short phalanges, cone epiphyses of the distal phalanges, pseudo-epiphysis in the middle phalanx of the second finger, and fifth finger clinodactyly with bone age 7.9 years (chronological age 8.7 years) (Fig. 1K). Femoral necks were asymmetrical with abnormal remodelling, shortening, and deformity (Fig. 1L). Endocrine assessment showed transient elevated serum IGF-I, increased plasma insulin concentration (Supplementary Table 1), and a normal GH response to clonidine. Breast development was relatively early but menarche was delayed and followed by oligomenorrhoea. Hair became progressively dry, sparse, and brittle (Fig. 1J), with increased scalp sensitivity.

Recombinant human growth hormone (rhGH) plus a GnRH analogue was administered from 10.1 to 11.6 years resulting in a small increase of height SDS, but was discontinued due to the poor growth response and development of acanthosis nigricans and hypertension. From 18 years onward, muscle cramps have been the major complaint, affecting limbs, abdominal muscles, tongue, and jaw. The electromyography (EMG) needle triggered painful vastus lateralis spasms, leading to prolonged continuous muscle activity (Fig. 1I). Cramps subsided with amitriptyline. Metabolic evaluation (Supplementary Table 1) showed progressive IR (treated with metformin), elevated serum triglycerides, and fatty liver.

#### Case 2

*Case 2* is a 25-year-old man, the only child of unrelated Portuguese parents. His mother is healthy and normal-statured. His father is short (−2.1 SDS), with a prematurely aged appearance, hearing impairment, obesity, premature loss of dentition, but normal intellectual ability (Fig. 2A). The proband was born with a low birthweight and showed poor postnatal growth (Fig. 2B and Table 1) and centripetal adiposity (BMI 2.5 SDS) (Fig. 2C, D, E, and F). Further clinical features include brachydactyly; mild fifth finger clinodactyly with broad, short nails; scattered depigmented patches on the abdomen; irregular café au lait patches on the lower back; joint hypermobility; supernumerary teeth; and mild acanthosis nigricans. Rapid, patchy hair loss was noted at age 25 years.

rhGH therapy from 9.5 to 10.5 years yielded no benefit and was discontinued due to excessive weight gain. The metabolic assessment showed extreme fasting hyperinsulinaemia without diabetes, reactive hypoglycaemia, fatty liver, and mildly elevated serum creatine kinase (Supplementary Table 2). For 13.6 years he has intermittently complained of muscle cramps. At 25 years old, he reported severe muscular pains, significantly worse than in his teenage years. These were spasmodic, associated with paraesthesia in the fingers, and were exacerbated by cold.

#### Case 3

*Case 3* is a 32-year-old Saudi Arab male born to parents who are first cousins and were diagnosed with DM2 at 42 years of age (Fig. 3A). The proband was born small for date (Table 1) and showed poor postnatal growth (Fig. 3B) and delayed developmental milestones (current IQ 68). Further clinical features include several facial dysmorphisms (detailed in Supplementary Information); brachydactyly; posteriorly rotated, low set ears; small, broad hands and feet with hypoplastic distal phalanges and nails; widely spaced first and second toes; single palmar creases; alopecia; and centripetal adiposity (Fig. 3B). A skeletal survey (Fig. 3C) revealed short femoral neck and phalanges, short left third metacarpal and metatarsal bone, hypoplastic distal phalanges and nails, and short, thick long bones.

GH deficiency was suspected and rhGH therapy was given from 8 years of age for 6 years, but information on serum IGF-I, GH stimulation testing, and growth response is unavailable. The metabolic assessment showed nuchal and axillary acanthosis nigricans, DM2, non-proliferative diabetic retinopathy, persisting fatty liver, hypercholesterolaemia, and hypertriglyceridaemia (Supplementary Table 3). For the borderline low plasma testosterone, no cause was found. At 26 years, muscle cramps in legs and chest on exertion and at rest were reported, with elevated serum creatinine kinase concentration. Muscle biopsy (Supplementary Fig. 1) showed nonspecific myopathic changes suggestive of a secondary neuropathic process.

### Laboratory investigations

Details of genetic analysis are presented in Supplementary information on genetic analyses. Biochemical investigations were undertaken in accredited hospital laboratories. The presented reference ranges are as provided by these laboratories, except for fasting plasma insulin, triglycerides, cholesterol, HDL, and LDL. Reference ranges for fasting insulin in prepubertal children (to 11 years) were from Peplies *et al*. (17), for pubertal adolescents from Ballerini *et al*. (18), and for young adults from Tohidi *et al*. (19). For plasma lipids, we used the recommendations of the Expert Panel on Integrated Guidelines for Cardiovascular Health and Risk Reduction in Children and Adolescents (20).

### Analysis of facial characteristics

Frontal facial photographs and clinical and genetic information of the three cases presented and eight previously reported (three from (3), single patients from (7, 9, 13, 14, 16)) were uploaded to the Face2Gene (FDNA Inc, Sunrise, FL, USA) platform. A ‘DeepGestalt‘ of the facial features of SOFT syndrome was generated, as previously reported for other syndromes (21).

## Results

In Case 1 a rare homozygous *POC1A* missense variant (c.649C>T, p.(Arg217Trp)) was found, as previously reported in a Chilean girl with SOFT syndrome (13). In Case 2 and his mother, the same p.(Arg217Trp) variant was identified in heterozygous form. A second rare heterozygous missense variant (c.370G>A), p.(Asp124Asn)) was detected in Case 2 but not his mother. The father was unavailable for study, but based on these findings the *POC1A* variants in the proband were deemed highly likely to be compound heterozygous. Case 3 harboured the same homozygous truncating variant in *POC1A* reported by Shaheen *et al*. in a Saudi family (3) (c.241C>T, p.(Arg81*)). Further details on these genetic variants are shown in Supplementary Table 4.

All known cases with SOFT syndrome and IR or DM2 are summarised in Supplementary Table 5. All six fully documented cases had acanthosis nigricans, insulin resistance, elevated triglycerides, and fatty liver. Data from three members of the large Arab pedigree reported by Shalev *et al*. (2), two of whom were reported to have DM2, are also shown. For these cases, the evaluation of plasma insulin, acanthosis nigricans, and fatty liver was unavailable, but serum triglycerides were increased (4.6, 6.2, and 5.5 mmol/L (reference <1.3 (20)) and HDL levels were low (0.9, 1.1, and 1.1 mmol/L, reference >1.2 (20), personal communication, Dr Shalev).

Supplementary Table 6 shows the anthropometric profile of all reported cases, stratified by the presence of IR. All except two patients without IR were younger than 15 years. In contrast, all patients with IR were older than 22 years. Auxological findings were similar between groups. Birth weight and length were low except for patients with the p.(Leu171Pro) variant (2). In contrast, head occipitofrontal circumference (OFC) at birth was normal in almost all patients resulting in relative macrocephaly. The average height was −5 to −6 SDS, with a wide range (−9 to −2 SDS), while OFC was relatively spared (mean approximately −3 SDS).

Based on analysis of facial characteristics of our patients and 8 reported previously, a general facial representation of patients with SOFT syndrome (DeepGestalt) was generated (Supplementary Fig. 2), featuring a prominent nose with a broad tip and broad mouth. Subjective inspection showed a triangular face in young children, less striking in older subjects. The syndrome is not yet recognised by the algorithms, which require further images for training (21).

## Discussion

This report conveys two main messages. Firstly, it solidifies dyslipidaemic IR and fatty liver as being associated with loss of *POC1A*function, showing this is not exclusive to pathogenic variants in exon 10. Second, it suggests that muscle involvement, likely secondary to neuronal dysregulation, is a novel phenotypic feature of SOFT syndrome.

Besides the dyslipidaemic IR in our three cases and three previously reported (7, 11, 16), we know of three cases with early-onset DM2 in a family reported in 2012 (2). Two of these were reported, with one further case diagnosed at 26 years old (Dr Shalev, personal communication). Nine cases of SOFT syndrome with reported dyslipidaemic IR, or 26% of all reported cases, are thus known. In most cases with IR, *POC1A* variants are outside exon 10, and anthropometric data do not discriminate cases with or without IR (Supplementary Table 6). We believe there is no basis to classify patients with biallelic *POC1A* variants and IR as having a specific subsyndrome as previously suggested (11). The prevalence of IR in SOFT syndrome would likely be higher if patients were biochemically screened from childhood onward. All but two previously reported cases without IR were younger than 15 years, while 8 of 9 cases with IR were adult at IR diagnosis (Supplementary Table 6), suggesting that IR development is age-dependent.

The mechanism linking dyslipidaemic IR to *POC1A* variants is unknown, but other forms of monogenic IR offer clues. Dyslipidaemia and fatty liver are common and severe in monogenic IR caused by adipose tissue defects, and the trajectory of dyslipidaemic IR in SOFT syndrome is reminiscent of lipodystrophies, where metabolic derangement commonly becomes clinically manifested peripubertally (22). In contrast, primary insulin signalling defects (in *INSR* or *PIK3R1*) do not result in dyslipidaemia or fatty liver (23, 24). Interestingly, several other genetic defects affecting the centrosome/primary cilium also feature dyslipidaemic IR, including Alström Syndrome (e.g. (25), caused by biallelic *ALMS1* variants (26) and Osteodysplastic Primordial Dwarfism of Majewski Type 2 (27), caused by biallelic *PCNT* variants (28). This suggests a possible unifying mechanism linking certain forms of centrosome dysfunction to IR, possibly mediated by effects on adipose tissue. Addressing this experimentally will be challenging due to the numerous functions of the centrosome, but the viability of mice with *Poc1a* deficiency, which recapitulate skeletal manifestations of SOFT syndrome (29), will permit future studies.

Regarding the question of how the loss of POC1A causes the broad clinical phenotype, we can only speculate. POC1A protein expression is nearly ubiquitous, so the pattern of tissue involvement cannot easily be explained by expression pattern alone. Given preliminary evidence of abnormal mitotic kinetics and perhaps shorter cilia in POC1A deficiency, and given recent evidence that cilia play a key role in adipocyte development* in vivo* (30), inefficient adipogenesis, or deranged kinetics of a mesenchymal stem cell pool, may impair the crucial function of adipose tissue in metabolic homeostasis. A similar phenomenon could be present in other tissues such as the epiphyseal growth plate, hair follicles, muscle, and gonads.

The effect of rhGH treatment in cases 1 and 2 was minimal, and in case 3 the low adult height achieved renders a positive effect of rhGH treatment unlikely. In case 1 this treatment coincided with worsened IR and increased blood pressure and in case 2 with increasing obesity. We therefore suggest that rhGH treatment is not indicated in SOFT syndrome.

To date, muscular cramps have not been included in SOFT syndrome (MIM # 614813), although reported in one Arab case (2). After we identified them as prominent complaints in our three cases, we approached a previously described patient with IR (7). She also reported severe muscle cramps in her hands, neck, abdomen, and legs from early childhood, usually at night, and more commonly in winter. A further patient described by Giorgio *et al*. (11) subsequently also complained of muscle cramps (Drs E. Rubino, A. Brusco; personal communication). Sica *et al*. (31) (MIM %600771) reported two brothers with short stature (130–132 cm), sparse scalp and absent body hair, low set ears, large noses, high-pitched voices, enlarged cardiac ventricles, and severe ‘undulating’ painful muscle spasms from 8 to 10 years. We believe these siblings likely had SOFT syndrome on clinical grounds. We therefore speculate that muscle cramps may be a common, albeit so far unrecognised, feature of the syndrome.

Muscle cramps and pain generally increased with exercise, associated with fasciculation-like twitches in limbs and elevated blood creatine kinase concentration. Electrophysiological evaluation, and some aspects of muscle biopsy, suggested a likely neurogenic origin. Further investigation and case descriptions are needed to elucidate the pathophysiology of neuromuscular involvement. Of note, the association between IR and muscle involvement is not unique to SOFT syndrome, however. The entity ‘acanthosis nigricans with muscle cramps and acral enlargement’ (MIM 200170), was described in 1980 (32, 33) and features of severe IR with phenytoin-responsive muscle cramps have been reported (33, 34). No features clearly conforming to SOFT syndrome were described. Other conditions such as some laminopathies and congenital generalised lipodystrophy type 4, feature myopathy and lipodystrophic IR (35).

The composite image of SOFT syndrome generates a step towards automated assistance to clinicians in making diagnoses on upload of a facial image and clinical features (21). Since SOFT syndrome is rare, the database could ultimately be of value in facilitating early diagnosis and screening for complications, however further images are required to train recognition algorithms fully. Although modern diagnostic procedures in high-income countries tend to use a hypothesis-free approach (e.g. next-generation sequencing techniques like exome sequencing (ES) and whole-genome sequencing in the near future), we believe that visual recognition of a facial phenotype remains important, particularly in countries where genetic testing is not available or reimbursed.

In conclusion, patients with SOFT syndrome often manifest severe dyslipidaemic IR and muscle cramps, independent of the position of the *POC1A* variant. After diagnosis, patients should be regularly screened for IR and muscle complaints. Further studies are needed to clarify the pathophysiology of these clinical features of SOFT syndrome.

## Supplementary Material

Supplementary Materials

## Declaration of interest

Prof Rob Semple is a Deputy Editor on the European Journal of Endocrinology Editorial board. Prof Rob Semple was not involved in the review or editorial process for this paper, on which he is listed as an author. The other authors declare no competing interests.

## Funding

This work was supported in part by the Wellcome Trust (grant number 210752/Z/18/Z to R K S).

## Author contribution statement

V M, I H-D, D A, J B, C C, K A, and R K S contributed by performing, interpreting and describing the clinical assessment of the patients. C de B advised on endocrine assessment, Y H was responsible for uploading and interpreting facial dysmorphology, and E B advised on the diagnosis of the muscle phenotype. F S A, M L, and R K S performed the genetic analyses. J M W coordinated the writing process. All authors contributed in data interpretation and various revisions of the manuscript and have approved the submitted manuscript.
