# Supplementary material for: Biallelic POC1A variants cause syndromic severe insulin resistance with muscle cramps
Source: Eur J Endocrinol. 2022 Mar 1;186(5):543–52. doi: 10.1530/EJE-21-0609 (PMC9010808; doi:10.1530/EJE-21-0609)
Supplement: Supplementary Materials [file supplementary_material.pdf]

Supplementary File to the paper "Biallelic POC1A variants cause syndromic severe insulin resistance with muscle cramps"

Veronica Mericq, Isabel Huang-Doran, Dhekra Al-Naqeb, Javiera Basaure, Claudia Castiglioni, Christiaan de Bruin, Yvonne Hendriks, Enrico Bertini, Fowzan S. Alkuraya, Monique Losekoot, Khalid Al-Rubeaan, Robert K. Semple, Jan M. Wit

Supplementary Information on clinical presentations

Case 1 is a 21.5-year-old Chilean woman born to healthy parents of normal height (father -0.6 SDS, mother -1.3 SDS) (1). Birthweight at 37 weeks' gestation was 1520 g (-4.4 SDS), length 39 cm (-5.5 SDS), and occipitofrontal circumference (OFC) 31 cm (-2.4 SDS) (1). Karyotype was normal. She showed normal psychomotor development but was admitted for failure to thrive at 2 years of age, when serum IGF-I was 101 ng/mL (-0.5 SDS) and IGFBP-3 2.3 mg/L (0.6 SDS) (2).

At 8.7 years endocrinological evaluation of severe short stature and bilateral hip pain was sought. Her height was 100.5 cm (-5.1 SDS), weight 14.3 kg (-4.4 SDS), Body Mass Index (BMI) 14.2 kg/m^2 (-1.2 SDS) (1) and OFC 47.5 cm (-3.5 SDS) (3). Arm span (100 cm) minus height was close to the mean for age (4). Cognitive function was normal. She had thin, slow-growing hair, a prominent forehead, deep-set eyes, hypoplastic nostrils, smooth philtrum, thin upper lip, light skin, café au lait macules, joint hyperlaxity, and broad hands and feet with broad thumbs/big toes. Radiographs showed short phalanges, cone epiphyses of the distal phalanges, pseudo-epiphysis in the middle phalanx of the second finger and fifth finger clinodactyly and bone age was 7.9 years**.** Femoral necks were asymmetrical with abnormal remodelling, shortening and deformity. Scintigraphy showed irregular contrast in the right hip with thickened epiphyseal growth plate and no increased osteoblastic activity, consistent with prior avascular necrosis. Serum IGF-I, and plasma insulin concentrations were increased (**Supplementary Table 1**). The serum growth hormone (GH) response to clonidine (11 ng/mL) was normal. Breast

development was at Tanner stage 3 at 9.3 years, consistent with plasma oestradiol, LH response to Leuprolide (**Supplementary Table 1**) and uterine size (3.9 cm). Ovaries remained small (0.6 and 1.0 mL) on ultrasonography.

At 10.8 years limbs showed greater shortening in proximal (<–4.4 SDS) than distal segments (-3.1 to -3.5 SDS). Sitting height/height ratio was normal (0.3 SDS) (5). Hands were small and wide but large in comparison to arms. Feet were small and in proportion to the legs. Three irregular café au lait macules were noted. From 10 years' old, hair became progressively dry, sparse and brittle, with increased scalp sensitivity.

Recombinant human growth hormone (rhGH) (0.05 mg/kg.d) was administered from 10.1 to 11.6 years in combination with a GnRH analogue. A small increase of height SDS (-5.5 to -5.2 SDS) was seen, but given this poor response, and development of acanthosis nigricans and hypertension (136/81mmHg at 11.8 years), rhGH treatment was discontinued. Hypertension was treated initially with enalapril. Menarche occurred at 15.3 years' old, followed by oligomenorrhoea.

At 18.7 years muscle cramps were the major complaint, affecting limbs, abdominal muscles, tongue and jaw. These had developed around the age of 2 years, and had since gradually increased in frequency and intensity, exacerbated by prolonged exercise but also present at rest, even overnight. Cramps were associated with serum creatine kinase concentrations more than ten times the upper limit of normal (**Supplementary Table 1**). Serum concentrations of calcium, phosphorus and magnesium were normal. Segmental muscle strength and muscle volumes were normal, with no osteotendinous contractures, nor muscle spasm after percussion.

At 21.5 years of age daily excruciating muscle spasms affected all skeletal muscles, lasting minutes to hours, triggered by movement and at rest. Spasms began with trunk and lower limb muscle stiffness, with co-contraction of agonist and antagonist muscles, sometimes with jerking involuntary limb

movements. Occasional mandibular and tongue spasms also occurred. Local warming tended to relieve pain. Nerve conduction studies were normal and concentric needle EMG showed a reduced recruitment pattern of motor units with polyphasic potentials of increased amplitude, indicating motor neuron involvement. Spontaneous muscle twitches, similar to fasciculations, were noted in limbs. The EMG needle triggered painful vastus lateralis spasms, leading to prolonged continuous muscle activity. Cramps subsided with amitriptyline.

Oligomenorrhoea persisted, associated with low serum oestradiol, high serum LH, biochemical hyperandrogenism and increased serum Anti-Mullerian Hormone concentration, which normalised on regular metformin (**Supplementary Table 1**). Pelvic ultrasound showed a uterine length of 5.5 cm, ovarian volumes of 12 and 4 ml, and multiple follicles of <10 mm. Adult height was 120 cm (-6.6 SDS), weight 29.6 kg, BMI 20.6 (0.3 SDS), OFC 50.0 cm (-4.0 SDS) and arm span 120 cm. Leg length was 60 cm, with a normal upper/lower segment ratio of 1.0.

Metabolic evaluation from 8.7 years old (**Supplementary Table 1**) showed progressive insulin resistance (IR), treated with metformin 850mg bid from 18.8 years, decreased to 425mg bid due to gastrointestinal symptoms. Serum triglycerides remained elevated from 11 years. Fatty liver was inferred from elevated aminotransferases and at 20.8 years, confirmed by ultrasonography. Blood pressure was well controlled on losartan 25 mg/day. Renal sonographic appearances were normal at 2 and 19.3 years, but at 21.5 years microalbuminuria and elevated blood urea nitrogen were recorded.

Case 2 is a 25-year-old man, the only child of unrelated Portuguese parents. His mother had a normal height (165 cm, 0.3 SDS) (6). His father was reported to be of short stature (162 cm, -2.1 SDS) (6), with a prematurely aged appearance, hearing impairment, obesity, and loss of dentition by 36 years. He was of normal intellectual ability.

The proband was born at 43 weeks' gestation with a birthweight of 2.45 kg [-3.2 SDS (7)]. A relatively large head and probably bony dysplasia were noted at birth, but skeletal surveys on two occasions during childhood failed to reveal a known dysplasia. Psychomotor development was normal. At 8.1 years height was 98 cm (-6.4 SDS), weight 21 kg (-1.6 SDS) and BMI 21.9 kg/m^2 (2.5 SDS) (8). Upper segment length was 54 cm [upper/lower segment ratio 1.23, equivalent to 3.5 SDS (4)] and arm span 93 cm [arm span minus height -1.3 SDS for age (9)]. Several small café au lait patches and joint hypermobility were noted. Brain MRI was normal. rhGH therapy from 9.5 to 10.5 years yielded no benefit, and was discontinued due to weight gain. Numerous dental procedures were required due to supernumerary teeth and dental caries, exacerbated by rapid jaw growth. Orthodontic assessment at age 12 revealed class III malocclusion. Nail growth was normal. Puberty onset was reportedly at 11 years old.

At 13.6 years old health was good except for muscle cramps. Height was 125.4 cm (-4.8 SDS), BMI 24.3 kg/m^2 (1.9 SDS) and OFC 53.8 cm (-1.2 SDS for age (8). Puberty was well advanced, with Tanner stage 3 genitalia and pubic hair, and testicular volumes 8 ml. Adiposity was centripetal but there was no frank lipodystrophy. Mild acanthosis nigricans was seen. There was brachydactyly and mild fifth finger clinodactyly with broad, short nails. There were scattered depigmented patches on the abdomen, and two small, irregular café au lait patches on the lower back. Muscle tone and limb reflexes were normal and no muscular hypertrophy was observed. Oral glucose tolerance testing revealed severe IR without diabetes, and reactive hypoglycaemia. Elevated serum ALT was consistent with fatty liver, and serum creatine kinase was mildly elevated (**Supplementary Table 2**). Serum calcium, phosphorus and magnesium concentrations were normal. Over the next 18 months cramps resolved, permitting vigorous activity, however acanthosis nigricans persisted.

At 22.3 years old, he was symptomatically well on no therapy but obesity had developed [height 127 cm (-7.2 SDS), weight 59.2 kg, BMI 36.7 kg/m^2]. Adiposity was centripetal with pronounced acanthosis nigricans. Arms and legs were short, but hair and nails were normal. Café au lait patches were unchanged. Biochemical evaluation demonstrated extreme fasting hyperinsulinaemia (**Supplementary Table 2**).

At 25 years old, he reported severe muscular pains, significantly worse than in teenage years. These were spasmodic, associated with paraesthesia in the fingers, and were exacerbated by cold. They were refractory to non-steroidal anti-inflammatory drugs, and limited activity, contributing to weight gain. No muscular hypertrophy was noted. He also described, for the first time, rapid, patchy hair loss occurring over several weeks in a non-androgenic distribution.

Case 3 is a 32-year-old Saudi Arab male presenting with short stature, intellectual disability, and type 2 diabetes mellitus (DM2). He was born at 39 weeks' gestation after a pregnancy complicated by intrauterine growth retardation. His parents are first cousins with 4 other healthy children. Both parents were diagnosed with DM2 at 42 years of age. At birth the proband was small for date [weight 1.8 kg (-2.8 SDS), length 45 cm (-3.0 SDS), OFC 33cm (-1.2 SDS)]. Developmental milestones were delayed from early childhood onwards. GH deficiency was suspected and rhGH therapy given from 8 years of age for 6 years, but information on serum IGF-I, GH stimulation testing and growth response is unavailable. Currently, he is semi-independent with an IQ of 68, has no secondary sexual characteristics, and has had alopecia since adolescence.

At 22 years his height was 135 cm (-5.8 SDS), weight 51.1 kg (-2.1 SDS), and BMI 28.2 kg/m^2. He had brachydactyly, posteriorly rotated, low set ears, small, broad hands and feet with hypoplastic distal phalanges and nails, partial alopecia, and centripetal adiposity. He exhibited a high forehead, hypotonia, joint hyperlaxity, brachycephaly, hypertelorism, broad upturned nose, long philtrum, short palpebral fissure, widely spaced first and second toes, and single

palmar creases. Skeletal survey revealed short femoral neck and phalanges, short left third metacarpal and metatarsal bone, hypoplastic distal phalanges and nails, and short, thick long bones. He had nuchal and axillary acanthosis nigricans and fatty liver, confirmed ultrasonographically. DM2 was diagnosed and managed with Metformin, Sitagliptin and Pioglitazone. On this regimen serum insulin was slightly increased, with C-peptide and adiponectin within normal limits (**Supplementary Table 3**).

At 25-26 years, lack of secondary sexual characteristics, plasma testosterone at or below the lower limit of normal and an empty sella on imaging prompted GnRH stimulation testing, which showed a normal FSH and LH response (10) (**Supplementary Table 3**). Three-weekly testosterone ester (250 mg) injections were prescribed, but compliance has been poor. Other pituitary axes were normal.

At 26 years, muscle cramps in legs and chest on exertion and at rest were reported, with elevated serum creatinine kinase concentration of 9702 U/L (reference 25-190 U/L). No muscular hypertrophy was noted. Cramp-Fasciculation Syndrome was suggested by electromyographic findings of rare fibrillation potentials and positive sharp waves, normal motor unit action, and cramps induced by exercise. Muscle biopsy (**Supplementary Figure 1**) showed nonspecific myopathic changes suggestive of a secondary neuropathic process. These included mild focal fibrosis, increased internal nuclei, occasional lobulation and splitting together with clusters of atrophic angular fibres and hypertrophic fibres, and focally increased internal nuclei. Cytochrome oxidase staining was uneven, with scattered fibres showing subsarcolemmal mitochondrial accumulation. Myosin heavy chain immunostaining showed dominance of type II fibres. Electron microscopy revealed scattered degenerating atrophic fibres and no clear mitochondrial abnormalities. Spinal MRI was normal. Symptoms abated gradually, and serum creatinine kinase concentration decreased to 300 U/L (**Supplementary Table 3**). Serum calcium, phosphorus and magnesium concentrations were always normal.

At 29 years severe hyperglycaemia was noted with HbA1c of 12.7%, mandating insulin therapy. Non-proliferative diabetic retinopathy was found with persisting fatty liver on ultrasonography and elevated serum aminotransferase concentrations. Hypercholesterolaemia and hypertriglyceridaemia were managed with Atorvastatin 20 mg daily.

Supplementary information on genetic analyses

POC1A variants identified are described with reference to RefSeq accession number NM_015426.4.

Case 1: The index and her parents were analysed in a diagnostic setting by exome sequencing at the Laboratory for Diagnostic Genome Analysis (LDGA), Department of Clinical Genetics, Leiden University Medical Centre. Genomic DNA was extracted from peripheral blood using the Chemagic Prime instrument (PerkinElmer, Waltham, MA, USA). Exomes were enriched with the SureSelect Clinical Research Exome V2 kit (Agilent Technologies, Santa Clara, CA, USA), followed by NovaSeq 6000 System sequencing (Illumina, San Diego, CA, USA). Variant analysis used a pipeline consisting of BWA, GATK and Moon software (http://www.diploid.com/moon) using the HPO terms for severe short stature. This resulted in the homozygous *POC1A* variant and no other plausible causal mutations were identified. No pathogenic mutations were identified in 95 myopathy-related genes in the laboratory of dr. Bertini (Italy) (11).

Case 2: Microarray revealed no pathogenic copy number changes. Exome sequencing of genomic DNA and variant calling were performed as part of the UK10K Project, as described previously (12). Raw sequence data is available from the European Genome-Phenome Archive (https://www.ebi.ac.uk/ega/home; accession EGAN00001015634). Two *POC1A* variants but no other plausible causal mutations were identified and were confirmed by Sanger sequencing.

Case 3: Woodhouse-Sakati syndrome was excluded by full sequencing of C2orf37 and autozygosity analysis. Exome sequencing was then undertaken and combined with the autozygome analysis as previously described (13, 14). No candidate variants in known myopathy genes were identified.

Supplementary Table 1. Selected laboratory findings in case 1

Age (years)	8.7	9.3	9.7	11	13	14.3	18.7	19.3	20.8	21.5	Reference range
Alanine aminotransferase (U/L)		32			52	**74**	**107**	37		**82**	<55 (21.5 yrs)
Aspartate aminotransferase (U/L)		**34**			**40**	**34**	**55**	20		33	5-34
Uric acid (µmol/L)										**571**	155-357
Creatine kinase (U/L)	111	163			123	111	**1038**	138		164	26-192 (<18 yrs), 29-168 (>18 yrs)
Creatine kinase-MB (U/L)	**31**	**60**			**31**	**23**	**45**				7-25 (<18 yrs), 0-25 (>18 yrs)
Total Cholesterol (mmol/L)		3.3			4.4	4.1	4.5	3.6	4.1	4.7-4.8	<4.4 (children, adolescents)<br><4.9 (young adults)
HDL Cholesterol (mmol/L)					**0.75**	**0.70**					>1.2
Triglycerides (mmol/L)				**2.6**	**2.6**	**2.1**	**4.3**	**2.7**	**2.0**	**3.9-5.9**	<1.0 (10-19 yrs), <1.3 (young adults)
OGTT glucose (mmol/L)											
Baseline		4.7			**6.2**	5.4	**5.9**	**5.6**	**5.6**	5.2	<5.6
120 mins					6.2	7.3	**9.1**			**13.9**	<7.8
OGTT insulin (pmol/L)											
Baseline	45			**192**	**733**		**348**			**304**	13-85 (8.7 yrs). 23-76 (Tanner 5)
120 mins	**650**				**2177**		**>2084**			**8084**	153-486 (8.7 yrs). 153-549 (Tanner 5)
IGF-1 (nmol/L)	**45**<br>(6-34)			**76**<br>(4-56)		28<br>(22-56)	26<br>(25-56)			**21**<br>(23-42)	
IGFBP-3 (mg/L)	5.2<br>(1.6-6.5)			6.6<br>(2.4-8.4)		7.0<br>(3.3-10)				5.7<br>(3.4-7.8)	
Oestradiol (pmol/L)			382				213			354, 268	<55 (prepubertal), 257-1101 (luteal)
LH (baseline/peak) (IU/L)			<0.6/14.2[1]				**22.1**			7.8	Prepubertal <0.6/<5. 2.8-14.0 (luteal)
FSH (baseline/peak) (IU/L)			3/17.7				4			2.2	Prepubertal <3. Luteal phase 1.4-5.5
Anti-Mullerian Hormone (pmol/L)										**1.6**	0.1-0.7
Testosterone (nmol/L)										**251**	37 - 197
SHBG (nmol/L)										**10**	11.7-137

[1]Baseline and peak levels (IRMA) at Leuprolide test, performed at 9.7 years. Abbreviations: FSH = Follicle-stimulating hormone; LH = luteinising hormone.

Laboratory results outside of the reference range are printed in **bold** print. OGTT = 75g oral glucose tolerance test .

Supplementary Table 2. Selected laboratory findings in case 2

**Age (years)**	**13.3**	**22.3**	**Reference range**
Alanine aminotransferase (U/L)	**60**	35	7-40
Total creatine kinase (U/L)	**1075**		42-163
Total cholesterol (mmol/L)	3.0	4.4	<4.4 to <4.9
LDL cholesterol (mmol/L)	1.8	0.29	<2.9 to <3.1
HDL cholesterol (mmol/L)	**0.96**	**0.83**	>1.2
Triglyceride (mmol/L)	0.6	**2.8**	<1.0 (10-19 yrs)<br><1.3 (18-21 yrs)
IGF-1 (nmol/L)	59.4		11.5-75.0
Testosterone (nmol/L)	14.0		8.0-32
Haemoglobin A1c (mmol/mol)	33		<42
OGTT glucose (mmol/L)<br>*0, 30, 60, 90, 120, 150, 180 mins*	4.0, 7.3, 6.7, 6.7, 6.8, 6.0, 2.7		Baseline <5.6
OGTT insulin (pmol/L)<br>*0, 30, 60, 90, 120, 150, 180 mins*	N/A, 2450, 2490, 2420, 3460, 2210, 410	**947**[1]	15-73

[1]Fasting level only at 22.3 years.

Abbreviations: NA = not available; OGTT = 75g oral glucose tolerance test.

Laboratory results outside of the reference range are printed in **bold** print.

Supplementary Table 3. Selected laboratory findings in case 3

**Age (years)**	**25**	**26**	**27**	**28**	**29**	**30**	**31**	**32**	**Reference range**
Alanine aminotransferase (U/L)		**102**	**44**	**59**	**71**	**42**	39	29	5-41
Aspartate aminotransferase (U/L)		**87**	28	30	30	23	26	16	12-37
Total creatine kinase (U/L)		**9702**	**307**	NA	**427**	**215**	**602**		25-190
Creatine kinase MB (U/L)		**122.1**							7-25
Total cholesterol (mmol/L)			**5.9**	**5.5**	**6.2**	**5.9**	**5.6**	**6.8**	<4.9
LDL cholesterol (mmol/L)			**4.3**	**3.8**	**4.3**	**3.6**	**3.7**	**3.5**	<3.1
HDL cholesterol (mmol/L)			1.2	**0.8**	**0.9**	**0.8**	**0.9**	**0.8**	>1.2
Triglycerides (mmol/L)			0.8	**2.0**	**2.5**	**3.1**	**2.1**	**5.5**	<1.3
Free T3 (pmol/L)			5.8				5.5		4.4-6.8
Free T4 (pmol/L)			17.0		17.1	16.7	18	17.8	12-22
Thyroid stimulating hormone (mIU/L)			1.6		1.7	2.2	1.8	2.1	2-5
Haemoglobin A1c (mmol/mol)			39	**64**	**82**	**96**	**71**	**95**	<42
Fasting capillary blood glucose (mmol/L)[1]						**15.7**			3.9-5.8
Insulin (pmol/L)[2]			**186**						15-73
C-peptide (pmol/L)			824						379-901
Adiponectin (µg/mL)			5.7						4-20[4]
Testosterone (nmol/L)	**6.1**	**9.5**	10.7			**8.0**	**6.7**		9.9-27.8
GnRH test: FSH (IU/L)[3]									
Baseline		3.3	4.4						0.7-7.2
Stimulated		7.9							0.5-10.5
GnRH test: LH (IU/L)[3]									
Baseline		7.5	5.1						1.8-8.4
Stimulated		38.9							7.4-54.2

[1]Patient was following by home glucose monitoring for blood sugar control. [2]On metformin 500mg twice daily, sitagliptin 100mg once daily and pioglitazone 15mg once daily. [3]Maximum of concentrations at 30, 60 and 90 minutes after injection of GnRH. Reference range from Bang et al, 2017 (10) [4]For males with BMI 25-30kg/m^2.

Abbreviations: FSH = Follicle-stimulating hormone; LH = luteinising hormone.

Laboratory results outside of the reference range are printed in **bold** print.

Supplementary Table 4. *POC1A* variants detected in this study

Nucleotide change (NM_015426.4)	Protein change	gnomAD (v2.1.1) MAF*	SIFT**	PolyPhen**	CADD score (Phred)	ClinVar	ACMG/AMP (Intervar)	Reference
c.649C>T	p.(Arg217Trp)	0.0028%	Not tolerated (p = 1.00)	Probably damaging (score 1.000; sensitivity 0.00; specificity 1.00)	25.5	Not present	VUS	13
c.370G>A	p.(Asp124Asn)	Not present	Not tolerated (p = 1.00)	Probably damaging (score 1.000; sensitivity 0.00; specificity: 1.00)	29.9	LP	LP	Not published
c.241C>T	p.(Arg81*)	0.0032%	-	-	37	1x P; 1x LP	P	3, 10, 29

*MAF = minor allele frequency; **Prediction of amino acid substitution.

Abbreviations: LP, Likely pathogenic; P, Pathogenic: VUS, variant of uncertain significance.

Supplementary Table 5. Clinical assessment parameters for reported cases with insulin resistance[a]

	**Cases 1-3[b]**	**Case 4**	**Case 5**	**Case 6**	**Case 7**	**Case 8**	**Case 9**
**First author and Date**	**Shalev *et al*, 2012**	**Chen *et al*, 2015**	**Giorgio *et al*, 2017**	**Majore *et al*, 2020**	**Present report**	**Present report**	**Present report**
Number of cases	3 out of 9	1	1	1	1	1	1
Ethnicity	Arab	Italian	Italian	Italian	Chilean	Portuguese	Arab
Height SDS	-7; -7; NR	-4.1	-4	-2.4	-7.2	-5.8	-5.8
IR/DM2	DM2; DM2; DM2	IR	IR/DM2	IR	IR	IR	IR/DM2
Age at diagnosis of DM2 or IR/gender	20/M; 24/M; 29/M	12.7/F	14/F	15/F	8/F	13/M	22/M
Hypertension	NR	No	NR	NR	+	NR	-
Hypertriglyceridaemia	+; +; +	+	+	+	+	+	-
Decreased HDL-chol	+, +, +	+			+	+	+
Centripetal adiposity	NR	+/-	+	+	-	+	+
Acanthosis nigricans (age noted)	NR	+ (8.5 yrs)	+ (7-8 yrs)	+ (13 yrs)	+ (11 yrs)	+ (13 yrs)	+ (<22 yrs)
Fatty liver	NR	+	+	+	+	+	+
PCOS	NA	+	irregular menses	+	+	NA	NA
Testicular failure	+; +; NR	NR	NA	NA	NA	-	+

[a]For genetic details, please see Supplementary Table 4. [b]The first 2 cases were reported by Shalev et al (2012). A third affected individual from this family (A-III-2) developed DM2 after publication. Data on serum lipids were kindly provided by Dr. Stavit Shalev.

Abbreviations: DM2, diabetes mellitus type 2; F, female; HDL-chol, HDL-cholesterol; IR, insulin resistance; M, male; NA, not applicable; NR, not reported; PCOS, polycystic ovary syndrome; +, present; -, absent

Supplementary Table 6. Anthropometric data in reported cases with SOFT syndrome

cDNA (NM-015426.4	Exon	Protein	N	Age, (sex)	Birth weight SDS	Birth length SDS	Birth OFC SDS	Height SDS	OFC SDS	Weight SDS	Reference
**Cases without severe insulin resistance**											
c.512T>C (Fam 1)	5	p.(Leu171Pro)	5	30 M@	-0.3*			-7.0	-3.0		(15, 16)
				23 F	0.5*	0.4*	2.7*	-6.0	-3.0		
				33 F				-9.0	-4.0		
				14 M@	-0.5*	-1.2*		-6.0	-4.0		
				0.75 M	-0.3*	-1.9*		-6.0	-2.5		
(Fam 2)			3	24 M				-7.0	-3.5		
				2.8 M	-0.3*	-0.8*	2.2*	-7.0	-1.7#		
				9 F	0.4*	-1.0*	0.3*	-8.0	-2.5		
c.241C>T (Fam 1)	3	p.(Arg81*)	3	6.0 F	-3.9	-4.7	-2.2	-6.7	-2.3	-5.0	(17)
				1.9 F	-3.8	-5.8	-2.2	-7.1		-6.3	
				0.3 M	-2.3	-3.0	-0.3^	-5.1	0.2^	-3.2	
(Fam 2)			1	6.0 M	-4.1			-7.1	-6.4	-6.3	
(Fam 3)			1	2.7 M	-6.6			-7.1	-3.3	-5.9	
c.358A>G	4	p.(Thr120Ala)	2	9/11.5 F	-4.0			-2.2	-0.3/-0.7&	-0.7/-1.3&	(18)
				6.5/8 M	-3.2			-5.0	-0.7/-1.3&	-3.1	
c.254del	3	p.(Leu85Alafs*22)	1	13.5 M	-4.0	-5.3	-1.2	-4.3			(19)
c.515G>A	5	p.(Trp172*)	1	1.1 M	-2.9	-4.5	-0.1	-7.2	-1.7	-4.8	
c.239C>T/<br>c.241C>T	3<br>3	p.(Ser80Phe)/<br>p.(Arg81*)	1	8.5 M	-4.3	-4.1		-6.7	-2.3	-6.0	(20)
c.491G>A	5	p.(Ser164Asn)	1	6.5 F	-3.1	-1.8	-1.6	-4.6	-0.3#	-4.0	(21)
c.649C>T	6	p.(Arg217Trp)	1	6.7 F	-2.2	-2.4	-0.8	-3.5	-1.4	-1.8	(22)
c.275+2T>G	Intr3	p.?	1	8.7 M	-3.0	-2.8	-0.2	-4.5	-4.1		(23)
c.64G>T (Fam 1)	2	p.(Val22Phe)	5	7.8 M	-3.2	-5.7	-2.7	-6.4	-6.0	-7.7	(24)
				2.0 F	-3.4	-3.7	-3.0	-6.3	-3.0	-11.7	
				3.8 M	-3.1	-4.4	-1.4	-6.5	-2.0	-6.9	
				3.8 M	-3.2	-4.4	-2.2	-7.5	-5.4#	-12.2	
				5.5 M	-2.7	-4.0	-1.4	-5.1	-6.0#	-6.2	
(Fam 2)			2	4.5 M	-3.6*	-5.0*	-0.4*	-5.5#	-1.4#	-7.2#	
				3.6 M	-1.9*	-3.7*	0.1*	-5.2#	-1.5#	-7.0#	
Mean (SD)			N=28	8.9 (19M,9F)	-2.7 (1.7)	-3.3(1.7)	-0.8 (1.5)	-6.1 (1.4)	-2.8 (1.8)	-5.9 (2.9)	

(range)				(0.3-30)	(-6.6;0.5)	(-5.8;0.4)	(-3.0;2.7)	(-9.0;-2.2)	(-6.4;0.2)	(-12.2;-1.3)	
**Cases with severe insulin resistance**											
c.1048del	10	p.(Gln350Argfs*4)	1	21.3 F	-3.1			-4.1			(12)
c.1048dup	10	p.(Gln350Profs*12	1	42 F				-4.0			(25)
c.884del/<br>c.1048del	9/10	p.(Val295Glyfs*59)/<br>p.(Gln350Argfs*4)	1	30 F	-1.6			-2.4			(26)
c.649C>T	6	p.(Arg217Trp)	1	8.8/22 F	-3.7	-4.3	-1.6	-5.3/-7.2	-4.7	-5.9	This report
c.370G>A/<br>c.649C>T	4/6	p. (Asp124Asn)/<br>p. (Arg217Trp)	1	32 M	-2.8	-3.0	-1.3^	-5.8	-1.2		This report
c.241C>T	3	p.(Arg81*)	1	32 M				-5.8		-2.1	This report
Mean (SD)<br>(range)			N=6	29.8 (7.6)<br>(21;42)	-2.8 (0.9)<br>(-3.7;-1.6)	-3.7<br>(-4.3;-3.0)	-1.5<br>(-1.6;-1.3)	-4.9 (1.7)<br>(-7.2;-2.4)	-3.0<br>(-4.7;-1.2)	-4.0<br>(-5.9;-2.1)	

@These patients developed diabetes mellitus type 2 at 20 and 26 years, respectively

*SDS of birth length, weight and OFC was calculated based on Swedish reference (7)

#SDS of these measures was calculated using Dutch references (27)

^Estimated based on percentile position

&The values at the oldest reported ages were used for calculating mean and SD

Other SDS values are copied from the original papers.

Abbreviations: F, female; M, male; OFC, occipitofrontal circumference; SDS, standard deviation score.

Supplementary Figure 1.

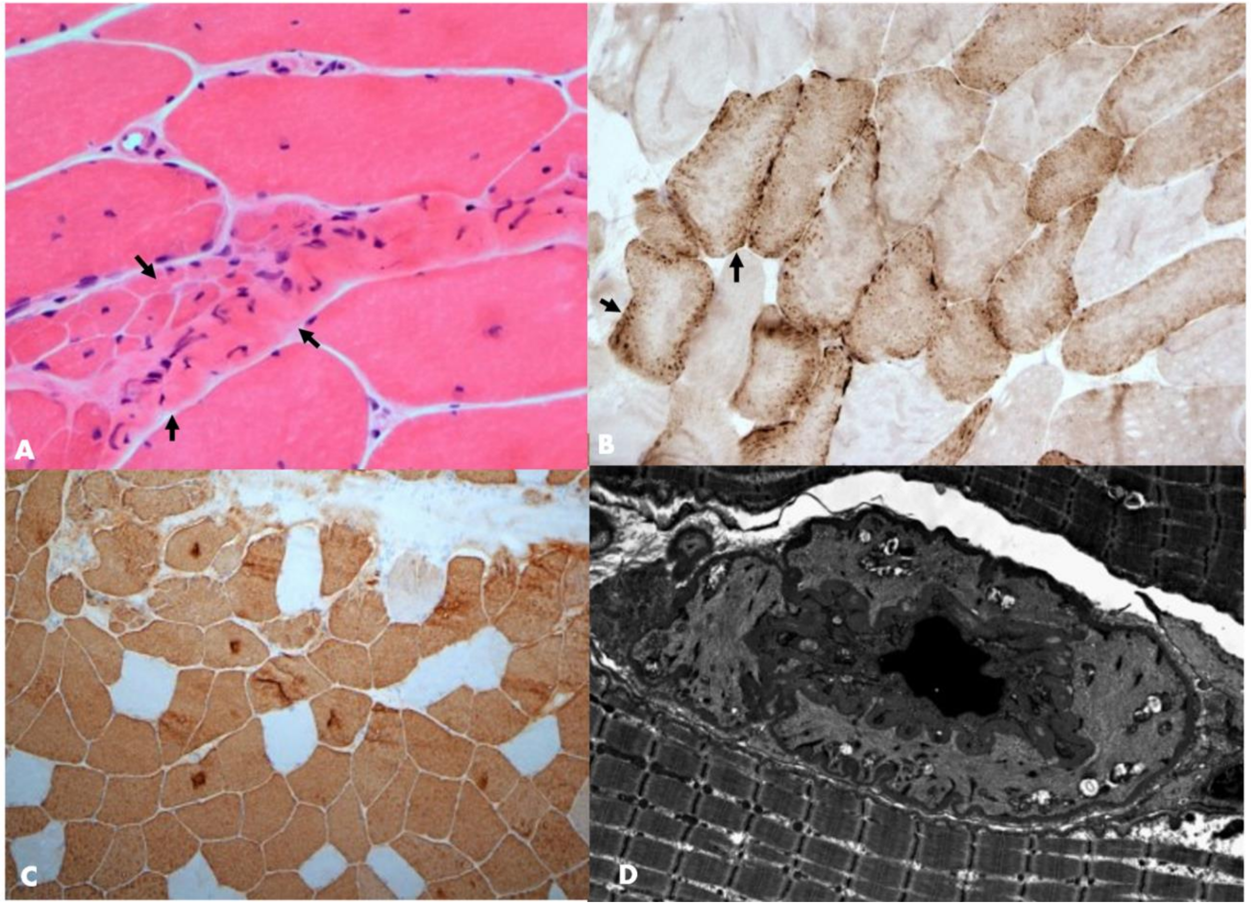

Muscle biopsy of patient 3. A: Haematoxylin and eosin staining reveals focal myopathic features, including variation in fibre size. Angular atrophic and hypertrophic fibres, focal prominent perimysial components and focal clumps of increased internal nuclei (black arrows) (X400). B: Cytochrome oxidase shows scattered fibres with subsarcolemmal mitochondrial accumulation (black arrows) (X200). C: Immunostaining for the fast class of myosin heavy chain highlights predominance of type II fibres (X100). D: Electron microscopy confirms nuclear abnormalities of the scattered degenerating atrophic angular fibres with nuclear clumps observed at light microscopy (X6000).

Supplementary Figure 2

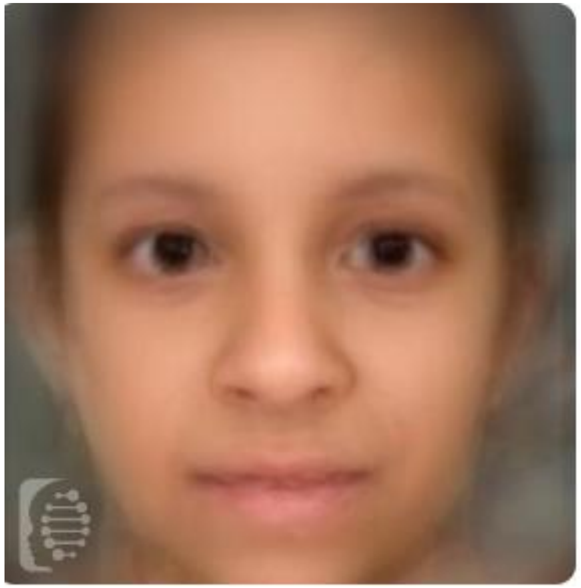

DeepGestalt of the face of patients with SOFT syndrome.

References

1. WHO Child Growth Standards based on length/height, weight and age. Acta Paediatr Suppl. 2006;450:76-85.
2. Elmlinger MW, Kuhnel W, Weber MM, Ranke MB. Reference ranges for two automated chemiluminescent assays for serum insulin-like growth factor I (IGF-I) and IGF-binding protein 3 (IGFBP-3). Clin Chem Lab Med. 2004;42(6):654-64.
3. Kuczmarski RJ, Ogden CL, Grummer-Strawn LM, Flegal KM, Guo SS, Wei R, Mei Z, Curtin LR, Roche AF, Johnson CL. CDC growth charts: United States. Adv Data. 2000(314):1-27.
4. Turan S, Bereket A, Omar A, Berber M, Ozen A, Bekiroglu N. Upper segment/lower segment ratio and armspan-height difference in healthy Turkish children. Acta Paediatr. 2005;94(4):407-13.
5. Fredriks AM, Van Buuren S, van Heel WJ, Dijkman-Neerincx RH, Verloove-Vanhorick SP, Wit JM. Nationwide age references for sitting height, leg length, and sitting height/height ratio, and their diagnostic value for disproportionate growth disorders. Arch Dis Child. 2005;90(8):807-12.
6. de Onis M, Onyango AW, Borghi E, Siyam A, Nishida C, Siekmann J. Development of a WHO growth reference for school-aged children and adolescents. Bull World Health Organ. 2007;85(9):660-7.
7. Niklasson A, Ericson A, Fryer JG, Karlberg J, Larwence C, Karlberg P. An Update of the Swedish Reference Standards for Weight, Length and Head Circumeference at Birth for Given Gestational Age (1977- 1981). Acta Paediatr Scand. 1991;80:756-62.
8. Wright CM, Williams AF, Elliman D, Bedford H, Birks E, Butler G, Sachs M, Moy RJ, Cole TJ. Using the new UK-WHO growth charts. BMJ. 2010;340:c1140.
9. Gerver WJM, Gkourogianni A, Dauber A, Nilsson O, Wit JM. Arm Span and Its Relation to Height in a 2- to 17-Year-Old Reference Population and Heterozygous Carriers of ACAN Variants. Horm Res Paediatr. 2020;93(3):164-72.
10. Bang AK, Nordkap L, Almstrup K, Priskorn L, Petersen JH, Rajpert-De Meyts E, Andersson AM, Juul A, Jorgensen N. Dynamic GnRH and hCG testing: establishment of new diagnostic reference levels. Eur J Endocrinol. 2017;176(4):379-91.
11. Fattori F, Fiorillo C, Rodolico C, Tasca G, Verardo M, Bellacchio E, Pizzi S, Ciolfi A, Fagiolari G, Lupica A, et al. Expanding the histopathological spectrum of CFL2-related myopathies. Clin Genet. 2018;93(6):1234-9.
12. Chen JH, Segni M, Payne F, Huang-Doran I, Sleigh A, Adams C, Consortium UK, Savage DB, O'Rahilly S, Semple RK, et al. Truncation of POC1A associated with short stature and extreme insulin resistance. J Mol Endocrinol. 2015;55(2):147-58.
13. Saudi Mendeliome G. Comprehensive gene panels provide advantages over clinical exome sequencing for Mendelian diseases. Genome Biol. 2015;16:134.
14. Monies D, Abouelhoda M, AlSayed M, Alhassnan Z, Alotaibi M, Kayyali H, Al-Owain M, Shah A, Rahbeeni Z, Al-Muhaizea MA, et al. The landscape of genetic diseases in Saudi Arabia based on the first 1000 diagnostic panels and exomes. Hum Genet. 2017;136(8):921-39.
15. Shalev SA, Spiegel R, Borochowitz ZU. A distinctive autosomal recessive syndrome of severe disproportionate short stature with short long bones, brachydactyly, and hypotrichosis in two consanguineous Arab families. Eur J Med Genet. 2012;55(4):256-64.

16. Sarig O, Nahum S, Rapaport D, Ishida-Yamamoto A, Fuchs-Telem D, Qiaoli L, Cohen-Katsenelson K, Spiegel R, Nousbeck J, Israeli S, et al. Short stature, onychodysplasia, facial dysmorphism, and hypotrichosis syndrome is caused by a POC1A mutation. Am J Hum Genet. 2012;91(2):337-42.
17. Shaheen R, Faqeih E, Shamseldin HE, Noche RR, Sunker A, Alshammari MJ, Al-Sheddi T, Adly N, Al-Dosari MS, Megason SG, et al. POC1A truncation mutation causes a ciliopathy in humans characterized by primordial dwarfism. Am J Hum Genet. 2012;91(2):330-6.
18. Koparir A, Karatas OF, Yuceturk B, Yuksel B, Bayrak AO, Gerdan OF, Sagiroglu MS, Gezdirici A, Kirimtay K, Selcuk E, et al. Novel POC1A mutation in primordial dwarfism reveals new insights for centriole biogenesis. Hum Mol Genet. 2015;24(19):5378-87.
19. Barraza-Garcia J, Ivan Rivera-Pedroza C, Salamanca L, Belinchon A, Lopez-Gonzalez V, Sentchordi-Montane L, del Pozo A, Santos-Simarro F, Campos-Barros A, Lapunzina P, et al. Two novel POC1A mutations in the primordial dwarfism, SOFT syndrome: Clinical homogeneity but also unreported malformations. Am J Med Genet A. 2016;170A(1):210-6.
20. Ko JM, Jung S, Seo J, Shin CH, Cheong HI, Choi M, Kim OH, Cho TJ. SOFT syndrome caused by compound heterozygous mutations of POC1A and its skeletal manifestation. J Hum Genet. 2016;61(6):561-4.
21. Mostofizadeh N, Gheidarloo M, Hashemipour M, Dehkordi EH. SOFT Syndrome: The First Case in Iran. Adv Biomed Res. 2018;7:128.
22. Saida K, Silva S, Solar B, Fujita A, Hamanaka K, Mitsuhashi S, Koshimizu E, Mizuguchi T, Miyatake S, Takata A, et al. SOFT syndrome in a patient from Chile. Am J Med Genet A. 2019;179(3):338-40.
23. Homma TK, Freire BL, Honjo Kawahira RS, Dauber A, Funari MFA, Lerario AM, Nishi MY, Albuquerque EV, Vasques GA, Collett-Solberg PF, et al. Genetic Disorders in Prenatal Onset Syndromic Short Stature Identified by Exome Sequencing. J Pediatr. 2019;215:192-8.
24. Al-Kindi A, Al-Shehhi M, Westenberger A, Beetz C, Scott P, Brandau O, Abbasi-Moheb L, Yuksel Z, Bauer P, Rolfs A, et al. A novel POC1A variant in an alternatively spliced exon causes classic SOFT syndrome: clinical presentation of seven patients. J Hum Genet. 2020;65(2):193-7.
25. Giorgio E, Rubino E, Bruselles A, Pizzi S, Rainero I, Duca S, Sirchia F, Pasini B, Tartaglia M, Brusco A. A syndromic extreme insulin resistance caused by biallelic POC1A mutations in exon 10. Eur J Endocrinol. 2017;177(5):K21-K7.
26. Majore S, Agolini E, Micale L, Pascolini G, Zuppi P, Cocciadiferro D, Morlino S, Mattiuzzo M, Valiante M, Castori M, et al. Clinical presentation and molecular characterization of a novel patient with variant POC1A-related syndrome. Clin Genet. 2020.
27. Fredriks AM, Van Buuren S, Burgmeijer RJ, Meulmeester JF, Beuker RJ, Brugman E, Roede MJ, Verloove-Vanhorick SP, Wit JM. Continuing positive secular growth change in The Netherlands 1955-1997. Pediatr Res. 2000;47(3):316-23.
